# Estimated pulse wave velocity and risk of new-onset heart failure in ICU patients with acute myocardial infarction: a multicenter cohort study

**DOI:** 10.3389/fmed.2026.1930499

**Published:** 2026-09-22

**Authors:** Huijian Wang, Shiyuan Li, Huabin He

**Affiliations:** 1 JiuJiang City Key Laboratory of Cell Therapy, Department of Cardiovascular Medicine, Jiujiang No. 1 People's Hospital, Jiujiang, Jiangxi, China; 2 Department of Cardiovascular Medicine, The Second Affiliated Hospital of Nanchang University, Nanchang, Jiangxi, China

**Keywords:** acute myocardial infarction, arterial stiffness, estimated pulse wave velocity, heart failure, risk stratification

## Abstract

**Background:**

To investigate the association between estimated pulse wave velocity (ePWV) and new-onset heart failure (HF) in ICU patients with acute myocardial infarction (AMI), and to evaluate its incremental predictive value.

**Methods:**

ICU patients with AMI were identified from the eICU-CRD cohort, and an independent real-world ICU cohort was used for external validation. New-onset HF was the primary outcome. Multivariable regression models were used to assess the association between ePWV and new-onset HF across different time periods. Restricted cubic spline analysis was performed to evaluate the dose-response relationship, and incremental value analysis was conducted to determine the added predictive value of ePWV beyond traditional risk models.

**Results:**

In the eICU-CRD cohort, the incidence of new-onset HF was 10.2%. After full adjustment, each 1-unit increase in ePWV was associated with a 13% higher risk of in-hospital heart failure (HR = 1.13, 95% CI: 1.09–1.18, *P* < 0.001), and the high-ePWV group had a significantly increased risk (HR = 1.84, 95% CI: 1.50–2.24, *P* < 0.001). In the validation cohort, the incidence of heart failure was 12.9%; each 1-unit increase in ePWV was associated with a 16% higher risk (OR = 1.16, 95% CI: 1.01–1.35, *P* = 0.046), and the high-ePWV group also showed increased risk (OR = 2.21, 95% CI: 1.02–4.78, *P* = 0.044). ePWV showed a nonlinear positive association with heart failure risk, and significantly improved model discrimination.

**Conclusions:**

Higher ePWV was independently and nonlinearly associated with in-hospital new-onset HF among ICU patients with AMI and improved risk discrimination beyond conventional clinical models. Its association with 1-year post-discharge HF in this high-risk population showed a consistent direction but requires further validation in larger prospective cohorts.

## Introduction

Acute myocardial infarction (AMI) is a common fatal cardiovascular disease characterized by myocardial necrosis caused by interruption of coronary blood flow (1). With the establishment of chest pain centers, the standardized use of electrocardiography and cardiac injury biomarker testing, and the implementation of early revascularization strategies, the early identification of AMI, the timeliness of reperfusion therapy, and clinical outcomes have improved substantially (2). However, despite progress in acute management, heart failure (HF) remains one of the most common and serious complications after AMI, imposing a substantial burden on patients and markedly affecting quality of life and prognosis (3). In contemporary AMI cohorts, the risk of new-onset HF has even exceeded that of recurrent myocardial infarction (4). Moreover, HF after AMI is strongly associated with adverse outcomes and is one of the strongest predictors of mortality, regardless of HF subtype (5, 6). HF represents one of the severe terminal stages of various cardiovascular diseases and is associated with high rates of disability and mortality (7). Therefore, identifying convenient and effective predictors for new-onset HF in this vulnerable population is important for improving risk stratification and enabling early intervention.

Arterial stiffness is a hallmark of vascular aging and has been shown to be positively associated with the risk of new-onset HF, independent of traditional risk (8, 9). Pulse wave velocity (PWV) is a widely accepted marker of arterial stiffness and is closely associated with cardiovascular risk and target organ damage (10). In European and American populations, carotid-femoral PWV (cfPWV) is considered the gold standard for assessing arterial stiffness (11), whereas in Asian populations, brachial-ankle PWV (baPWV) is more widely used because of its broader evidence base (12). However, both cfPWV and baPWV require dedicated equipment and standardized measurement procedures, limiting their application in routine clinical practice and large-scale retrospective studies. To address this limitation, Greve et al. developed estimated pulse wave velocity (ePWV), a simple index derived from age and mean blood pressure (13). Subsequent studies have shown that ePWV has predictive performance comparable to that of cfPWV and baPWV. A secondary analysis of the SPRINT trial demonstrated good agreement between ePWV and cfPWV in predicting cardiovascular events (14), and a prospective study in China showed that ePWV outperformed baPWV in predicting all-cause and cardiovascular mortality in patients with hypertension (15). In addition, ePWV has been identified as an independent predictor of new-onset HF in the general population (16). Beyond its role as a surrogate marker of arterial stiffness, ePWV has also demonstrated prognostic value for adverse clinical outcomes. Previous studies showed that its association with mortality remained significant after adjustment for conventional cardiovascular risk factors and established risk scores, while the addition of ePWV further improved risk discrimination and reclassification (17). More recent evidence also supports the independent association of ePWV with long-term clinical outcomes, highlighting its potential utility as a simple marker for cardiovascular risk stratification (18).

However, direct evidence regarding the value of ePWV for predicting new-onset HF among ICU patients with AMI remains limited. Therefore, this study aimed to investigate the predictive value of ePWV for new-onset HF among ICU patients with AMI using a large public critical care database and to further validate the robustness of the findings in an independent real-world ICU cohort. Because ePWV can be derived from routinely available age and blood pressure measurements, it may be particularly useful for early risk stratification when direct arterial stiffness assessment is unavailable.

## Methods

### Study population

This study used the eICU-CRD database and a cohort from the Second Affiliated Hospital of Nanchang University as data sources. The eICU-CRD is a publicly available critical care database established by Philips Healthcare, containing de-identified data from more than 200,000 ICU admissions at 208 U.S. hospitals between 2014 and 2015. The authors completed the required training and obtained access to the database (certification no. 63644491). Clinical data from patients with AMI admitted to the ICU of the Second Affiliated Hospital of Nanchang University between 2024 and 2025 were also collected. The study was approved by the hospital's Ethics Committee (approval no. IIT-2025-553). As this was a retrospective observational study with anonymized data, informed consent was waived.

The inclusion and exclusion criteria for both cohorts were as follows: (1) age >18 years; (2) ICU admission with a diagnosis of AMI; (3) for patients with multiple hospitalizations, only data from the first hospitalization were extracted; (4) patients with a prior history of HF were excluded; (5) patients with missing admission blood pressure data were excluded; (6) patients diagnosed with cardiogenic shock within 24 h of admission were excluded; and (7) patients with admission blood pressure >180/120 mmHg were excluded. The patient selection flowchart is shown in Supplementary Figure S1. Ultimately, 4,498 patients were included in the eICU-CRD cohort, and 255 patients were included in the cohort from the Second Affiliated Hospital of Nanchang University.

### Data collection and definitions

For the eICU-CRD cohort, baseline data were extracted using Structured Query Language (SQL) in PostgreSQL (version 16.0). Variables included general characteristics, vital signs, laboratory parameters, and comorbidities. Laboratory data were defined as the first measurements obtained within 24 h of admission. Continuous variables with less than 20% missing data were imputed using multiple imputation by chained equations (MICE), generating 10 imputed datasets with predictive mean matching. ePWV, HF outcome and event time, and categorical variables were not imputed but were included as predictors in the imputation models where appropriate. Detailed patterns of missingness are provided in Supplementary Table S1. Convergence was assessed based on between-chain variability and the stability of imputed values during later iterations (Supplementary Table S2). Diagnoses of AMI, chronic kidney disease, atrial fibrillation, hypertension, diabetes mellitus, and heart failure were identified using ICD-9/ICD-10 codes. Detailed HF diagnostic codes and data sources are provided in Supplementary Table S3. Medical history was retrieved through the pasthistory table. For HF ascertainment, patients with documented pre-existing or admission-present HF were first excluded. Among the remaining patients, HF events were identified from the diagnosis table using prespecified HF diagnostic codes. When multiple HF records were available, the earliest qualifying record was defined as the first HF event, and the corresponding diagnosisoffset was used to determine event timing relative to ICU admission. Premature coronary artery disease (PCAD) is defined as coronary artery disease with onset at age <55 years in men and <65 years in women. For the cohort from the Second Affiliated Hospital of Nanchang University, the same variables were collected, with additional procedural data including PCI status and Gensini score. The exposure variable was ePWV, which was derived by integrating age and mean arterial pressure (MAP). In this study, ePWV was calculated according to the formula reported by Greve et al: ePWV = 9.587−0.402 × age + 4.560 × 10^^−3^ × age^2^−2.621 × 10^^−5^ × age^2^ × MAP + 3.176 × 10^^−3^ × age × MAP−1.832 × 10^^−2^ × MAP (13). Blood pressure was defined as the first non-invasive blood pressure measurement recorded at admission in both cohorts, and invasive arterial-line measurements were not used. A higher ePWV value indicates a greater degree of arterial stiffness. For analytical purposes, ePWV was categorized in two ways: first, into three groups according to tertiles; second, into two groups based on the optimal cutoff determined by the Youden index.

### Clinical outcomes

The clinical outcome of this study was new-onset HF. In the eICU-CRD cohort, follow-up covered the entire hospitalization period. In the cohort from the Second Affiliated Hospital of Nanchang University, follow-up included the entire hospitalization period and 1-year post-discharge follow-up.

### Statistical analysis

The normality of continuous variables was assessed using the Shapiro–Wilk test. Normally distributed variables were expressed as mean ± standard deviation (SD) and compared using the independent-samples t test, whereas non-normally distributed variables were presented as median (first quartile, third quartile) and compared using the Mann–Whitney U test. Categorical variables were expressed as counts and percentages and compared using Pearson's chi-square test. Statistical analyses were first performed in the eICU-CRD cohort and then independently validated in the real-world cohort from the Second Affiliated Hospital of Nanchang University. Kaplan–Meier (KM) cumulative incidence curves were plotted to assess the cumulative risk of post-myocardial infarction HF during hospitalization and across different ePWV levels. Potential confounders were screened using least absolute shrinkage and selection operator (LASSO) regression, with the penalty parameter selected by cross-validation (Supplementary Figure S2). Multicollinearity was assessed using variance inflation factors (VIFs), with a VIF <5 considered to indicate the absence of substantial linear multicollinearity (Supplementary Table S4). Based on the LASSO-selected covariates and clinical relevance, three sequential models were constructed. Model 1 was unadjusted. Model 2 was adjusted for sex, BMI, and heart rate. Model 3 was further adjusted for albumin, neutrophil percentage, serum creatinine, hemoglobin, chronic kidney disease, atrial fibrillation, and ST-segment elevation myocardial infarction. Cox proportional hazards regression models were then used to evaluate the association between ePWV and post-myocardial infarction HF. Restricted cubic spline (RCS) analysis and threshold effect analysis were further performed to explore the potential linear or nonlinear relationship between ePWV and the outcome. To evaluate the incremental predictive value of ePWV, we constructed a common clinical base model that included the covariates from the fully adjusted model, excluding age, MAP, and ePWV. Age, MAP, and ePWV were then added separately to the same base model, yielding three directly comparable extended models: base model + age, base model + MAP, and base model + ePWV. Using the common base model as the reference, the incremental predictive performance of each variable was assessed by changes in the time-dependent area under the receiver operating characteristic curve (AUC), continuous net reclassification improvement (NRI), and integrated discrimination improvement (IDI). Subgroup analyses stratified by sex, BMI, premature coronary artery disease (PCAD), hypertension, myocardial infarction type, and atrial fibrillation were also performed to assess the consistency of the predictive value of ePWV across subgroups and to test for interaction effects. In the cohort from the Second Affiliated Hospital of Nanchang University, because the exact timing of new-onset HF during hospitalization was unavailable, logistic regression analysis was used to assess the association between ePWV and in-hospital post-myocardial infarction HF. PCI status and Gensini score were further included in the models, and subgroup analyses additionally considered the severity of coronary artery disease. Finally, sensitivity analyses were performed. First, participants who developed HF within the first 24 h after admission were excluded, and the associations were re-evaluated using Cox proportional hazards regression. Second, with discharge and death treated as competing events, Fine–Gray subdistribution hazard models were fitted to account for competing risks. Third, vasoactive/inotropic drug use before or at the first non-invasive blood pressure measurement was additionally adjusted for in the fully adjusted models. All statistical analyses were performed using R software version 4.3.1, and a two-sided *P* < 0.05 was considered statistically significant.

## Results

### Baseline characteristics

A total of 4,498 ICU patients with AMI were included in the eICU-CRD cohort, with a median age of 64 years; 66.2% were male, and 59.9% had ST-segment elevation myocardial infarction (Table 1). Compared with the non-HF group, patients in the HF group were older and had a higher proportion of women and ST-segment elevation myocardial infarction, higher ePWV levels, lower blood pressure, and a faster heart rate. They also had higher white blood cell count, serum creatinine, blood urea nitrogen, and serum potassium levels, but lower hemoglobin and albumin levels. In addition, the prevalences of chronic kidney disease, atrial fibrillation, hypertension, and diabetes mellitus were higher in patients who developed HF. A total of 255 ICU patients with AMI were included in the cohort from the Second Affiliated Hospital of Nanchang University. Compared with the non-HF group, patients in the HF group were older, had higher Gensini scores, a lower rate of PCI, higher blood urea nitrogen levels, lower hemoglobin and albumin levels, and a higher prevalence of chronic kidney disease (Supplementary Table S5).

**Table 1:** Baseline characteristics of the study population in the eICU-CRD cohort stratified by outcome.

Variables	Overall (*n* = 4498)	No new-onset HF (*n* = 4036)	New-onset HF (*n* = 462)	*P*-value
Age, years	64.0 [54.0, 73.0]	63.0 [54.0, 72.0]	72.0 [63.2, 80.0]	< 0.001
Male, *n* (%)	2980 (66.25)	2744 (67.34)	236 (55.79)	< 0.001
Height, cm	172.7 [165.0, 178.0]	172.7 [165.1, 180.0]	167.6 [160.0, 175.3]	<0.001
Weight, kg	83.9 [72.4, 98.5]	84.4 [72.6, 98.9]	79.3 [67.6, 93.4]	<0.001
BMI, kg/m^2^	28.4 [25.0, 32.6]	28.4 [25.1, 32.6]	28.2 [24.1, 32.3]	0.263
STEMI, *n* (%)	2696 (59.9)	2354 (58.3)	342 (74.0)	< 0.001
PCAD, *n* (%)	906 (20.1)	861 (21.3)	45 (9.7)	< 0.001
ePWV	9.9 [8.5, 11.6]	9.7 [8.4, 11.5]	10.9 [9.5, 12.7]	< 0.001
Vital signs				
Temperature, ℃	36.6 [36.4, 36.9]	36.6 [36.4, 36.9]	36.7 [36.4, 37.0]	0.2
Heart rate, beats/min	80.0 [69.0, 94.0]	79.0 [68.0, 92.0]	95.5 [80.0, 108.0]	<0.001
SBP, mmhg	128.0 [113.0, 145.0]	129.0 [114.0, 145.0]	125.5 [109.0, 143.0]	0.004
DBP, mmhg	76.0 [66.0, 85.0]	76.0 [66.0, 86.0]	71.0 [59.0, 84.0]	<0.001
MAP, mmhg	93.3 [83.0, 104.3]	93.7 [66.0, 86.0]	89.3 [75.4, 102.7]	<0.001
SpO2, %	98.0 [95.0, 99.0]	98.0 [96.0, 99.0]	97.0 [94.0, 99.0]	<0.001
Respiratory rate, beats/min	18.0 [16.0, 21.0]	18.0 [16.0, 21.0]	20.0 [17.0, 25.0]	<0.001
Laboratory indicators				
WBC, k/uL	10.6 [8.2, 13.6]	10.4 [8.2, 13.4]	12.0 [8.8, 15.7]	<0.001
RBC, k/uL	4.5 [4.0, 4.9]	4.6 [4.1, 5.0]	4.1 [3.6, 4.7]	<0.001
Platelets, k/uL	223.0 [180.0, 272.0]	223.0 [181.0, 270.0]	223.0 [170.0, 291.0]	0.962
Hemoglobin, g/L	137.0 [122.0, 149.0]	138.0 [124.0, 150.0]	124.0 [106.0, 140.0]	<0.001
Neu, %	72.0 [60.1, 81.0]	71.0 [60.0, 80.0]	78.0 [69.0, 86.0]	<0.001
Mon, %	7.2 [5.5, 9.0]	7.2 [5.7, 9.0]	7.0 [5.0, 9.0]	0.004
Lym, %	17.1 [10.0, 27.0]	18.0 [11.0, 28.0]	11.8 [6.0, 19.0]	<0.001
Albumin, mg/L	36.0 [32.0, 40.0]	36.0 [33.0, 40.0]	33.0 [28.0, 37.0]	<0.001
Creatinine, μmol/L	88.4 [70.7, 109.6]	86.6 [70.7, 106.1]	106.1 [82.2, 157.8]	<0.001
BUN, mg/dL	17.0 [13.0, 23.0]	16.0 [13.0, 21.0]	24.0 [17.0, 37.0]	<0.001
ALT, U/L	30.0 [20.0, 46.0]	30.0 [20.0, 46.0]	28.0 [18.0, 51.8]	0.556
AST, U/L	36.0 [23.0, 79.0]	36.0 [23.0, 78.0]	40.0 [26.0, 86.0]	0.018
Glucose, mmol/L	7.4 [6.2, 10.1]	7.4 [6.2, 9.8]	8.7 [6.6, 13.7]	<0.001
Tg, mmol/L	1.3 [0.9, 2.0]	1.3 [1.0, 2.0]	1.2 [0.9, 1.7]	<0.001
Tc, mmol/L	4.2 [3.4, 4.9]	4.2 [3.4, 5.0]	3.7 [3.1, 4.5]	<0.001
LDL, mmol/L	2.4 [1.8, 3.1]	2.4 [1.8, 3.1]	2.0 [1.5, 2.7]	<0.001
HDL, mmol/L	1.0 [0.8, 1.2]	1.0 [0.8, 1.2]	1.0 [0.8, 1.3]	0.079
Sodium, mmol/L	138.0 [135.0, 140.0]	138.0 [136.0, 140.0]	137.0 [134.0, 140.0]	<0.001
Potassium, mmol/L	4.0 [3.7, 4.3]	4.0 [3.7, 4.3]	4.2 [3.8, 4.6]	<0.001
BNP, pg/ml	313.0 [87.0, 1140.0]	271.0 [80.0, 998.3]	1160.0 [434.0, 5350.5]	<0.001
Comorbidities, *n* (%)				
CKD	309 (6.8)	236 (5.8)	73 (15.8)	< 0.001
AF	413 (9.2)	301 (7.5)	112 (24.2)	< 0.001
HTN	2614 (58.1)	2306 (57.1)	308 (66.6)	< 0.001
DM	1027 (22.8)	884 (21.9)	143 (30.9)	< 0.001

Continuous variables are presented as median [Q1, Q3].

BMI, body mass index; STEMI, ST-segment elevation myocardial infarction; PCAD, premature coronary artery disease; ePWV, estimated pulse wave velocity; SBP, systolic blood pressure; DBP, diastolic blood pressure; MAP, mean arterial pressure; SpO2, oxyhemoglobin saturation; WBC, white blood cell; RBC, red blood cell; BUN, blood urea nitrogen; ALT, alanine aminotransferase; AST, aspartate aminotransferase; Tg, triglyceride; Tc, total cholesterol; LDL, low-density lipoprotein; HDL, high-density lipoprotein; BNP, B-type natriuretic peptide; CKD, chronic kidney disease; AF, atrial fibrillation; HTN, hypertension; DM, diabetes mellitus.

### KM analysis of ePWV and new-onset HF

The KM cumulative risk curves showed that, in the eICU-CRD cohort, the cumulative risk of in-hospital new-onset HF in critically ill patients with AMI increased progressively with higher ePWV levels, regardless of whether ePWV was categorized into tertiles or dichotomized using the optimal cutoff value. The differences between groups were statistically significant in both analyses (log-rank *P* < 0.001) (Figure 1). This pattern remained unchanged even after excluding HF events occurring within the first 24 h of admission, with significant between-group differences (log-rank *P* < 0.001) (Supplementary Figure S3). In the cohort from the Second Affiliated Hospital of Nanchang University, the high-ePWV group showed an overall trend toward a higher risk of HF within 1 year after discharge, although the between-group difference did not reach statistical significance (log-rank *P* = 0.074) (Supplementary Figure S4).

**Figure 1:**
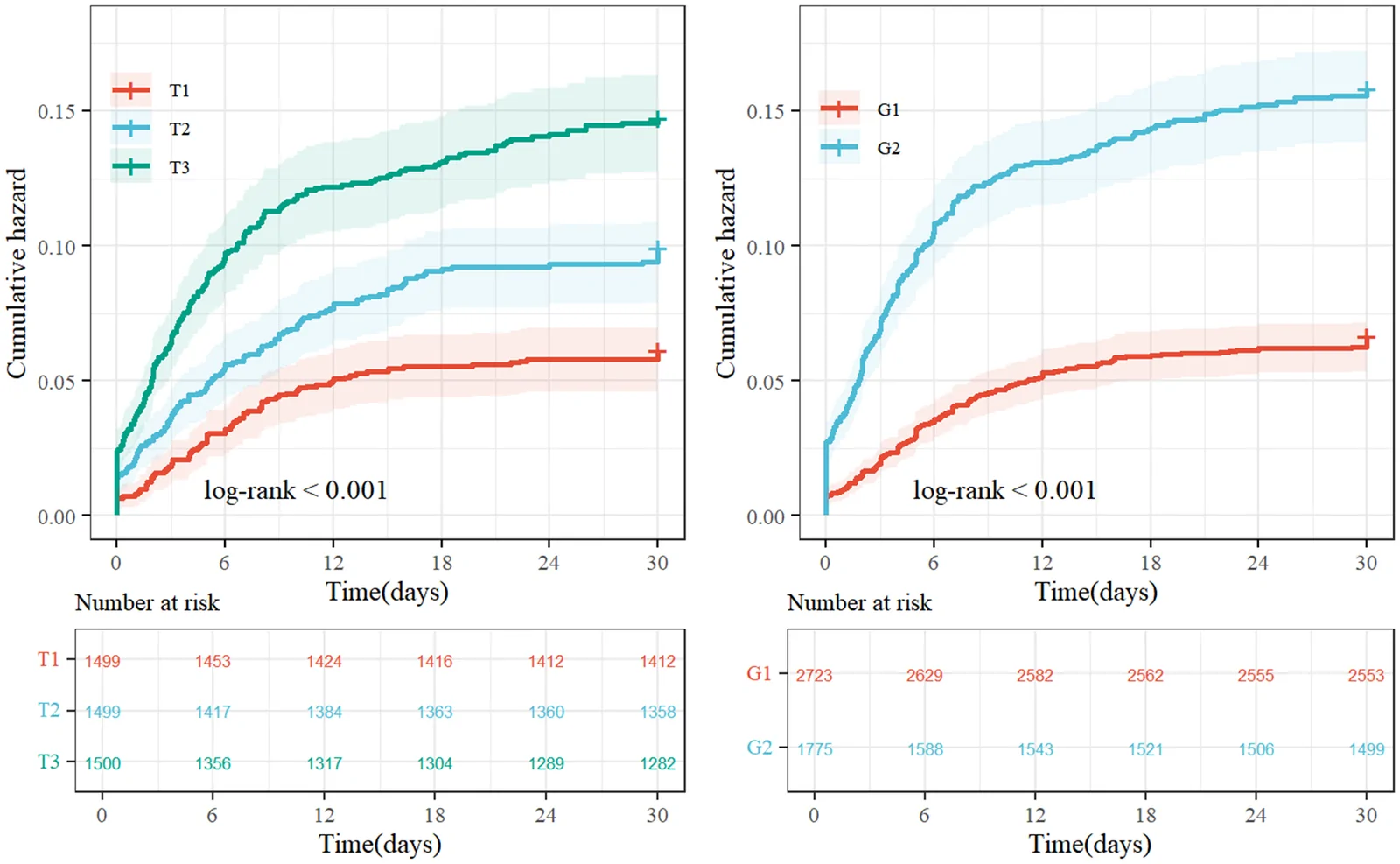
KM cumulative curves (left: ePWV tertile groups; right: ePWV groups by optimal cutoff).

### Association between ePWV and new-onset HF

Table 2 shows that, among ICU patients with AMI in the eICU-CRD cohort, higher ePWV was significantly associated with an increased risk of new-onset HF. After full adjustment, each 1-unit increase in ePWV was associated with a 13% higher risk of in-hospital new-onset HF (HR = 1.13, 95% CI: 1.09–1.18, *P* < 0.001). When categorized by tertiles, the risks in both the T2 and T3 groups were higher than that in the T1 group, showing a dose-response trend. When dichotomized using the optimal cutoff value, the high-ePWV group still had a significantly higher risk than the low-ePWV group (HR = 1.84, 95% CI: 1.50–2.24, *P* < 0.001). In the external cohort, the association between higher ePWV and in-hospital new-onset HF was directionally consistent with that observed in the eICU-CRD cohort. After further adjustment for PCI status and Gensini score, the association remained statistically significant; however, given the limited number of HF events, these findings should be interpreted as supportive rather than definitive evidence (Supplementary Table S6). As a continuous variable, each 1-unit increase in ePWV was associated with a 16% higher risk (OR = 1.16, 95% CI: 1.01–1.35, *P* = 0.046). When categorized by tertiles, only the T3 group showed a significantly increased risk, with an overall increasing trend across groups. When dichotomized using the optimal cutoff value, the high-ePWV group still had a significantly increased risk (OR = 2.21, 95% CI: 1.02–4.78, *P* = 0.044). For the 1-year follow-up after discharge, univariable analysis showed that ePWV was associated with an increased risk, whereas after full adjustment for confounders, only an overall trend toward higher risk was observed and the association did not reach statistical significance (Supplementary Table S7). In addition, the RCS curves indicated a nonlinear positive association between ePWV and the risk of new-onset HF among ICU patients with AMI (overall *P* < 0.001; nonlinear *P* = 0.022) (Figure 2). Further threshold effect analysis showed no significant threshold effect in the fully adjusted model (likelihood ratio test *P* = 0.142) (Supplementary Table S8).

**Table 2:** Association of ePWV with in-hospital new-onset heart failure in critically ill patients with AMI in the eICU-CRD cohort.

ePWV	All	Events	Model 1		Model 2		Model 3	
			HR (95% CI)	*P*-value	HR (95% CI)	*P*-value	HR (95% CI)	*P*-value
In-hospital								
Continuous	4498	462 (10.2)	1.22 (1.17–1.27)	*P* < 0.001	1.20 (1.15–1.25)	*P* < 0.001	1.13 (1.09–1.18)	*P* < 0.001
Tertiles								
T1 (< 8.93)	1499	92 (6.1)	Ref.		Ref.		Ref.	
T2 (8.93–10.98)	1499	149 (9.9)	1.63 (1.24–2.14)	*P* < 0.001	1.60 (1.22–2.10)	*P* = 0.001	1.45 (1.10–1.90)	*P* = 0.008
T3 (> 10.98)	1500	221 (14.7)	2.50 (1.94–3.22)	*P* < 0.001	2.33 (1.80–3.00)	*P* < 0.001	1.67 (1.28–2.17)	*P* < 0.001
*P* for trend				*P* < 0.001		*P* < 0.001		*P* < 0.001
Cutoff-based								
Q1 (≤ 10.54)	2723	181 (6.6)	Ref.		Ref.		Ref.	
Q2 (> 10.54)	1775	281 (15.8)	2.56 (2.11–3.11)	*P* < 0.001	2.44 (2.01–2.97)	*P* < 0.001	1.84 (1.50–2.24)	*P* < 0.001

Model 1: unadjusted.

Model 2: adjusted for gender, BMI, and heart rate.

Model 3: adjusted for gender, BMI, heart rate, albumin, neutrophil percentage, creatinine, BNP, hemoglobin, CKD, AF, and STEMI.

**Figure 2:**
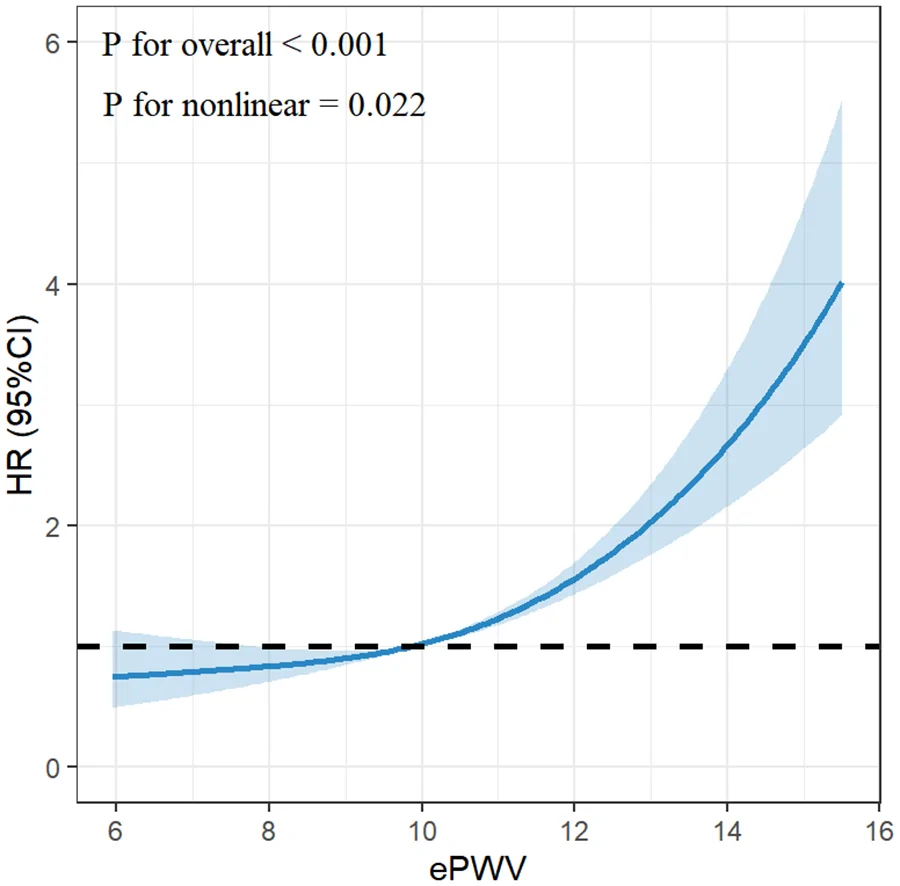
RCS curve in the eICU-CRD cohort.

### Incremental value analysis

The results of the incremental predictive value analysis are shown in Table 3. Using the same clinical base model as the reference, age, MAP, and ePWV were separately added to construct three directly comparable models. The time-dependent AUC of the base model was 0.817. After the addition of age, MAP, and ePWV, the AUC increased to 0.823, 0.821, and 0.828, respectively (all *P* < 0.001 versus the base model). The corresponding IDI values were 0.011 (95% CI: 0.001–0.021, *P* = 0.029), 0.008 (95% CI: 0.001–0.015, *P* = 0.033), and 0.014 (95% CI: 0.002–0.026, *P* = 0.024), while the NRI values were 0.127 (95% CI: 0.051–0.203, *P* < 0.001), 0.109 (95% CI: 0.044–0.174, *P* < 0.001), and 0.162 (95% CI: 0.107–0.239, *P* < 0.001), respectively. Thus, although all three variables improved predictive performance when separately added to the same base model, ePWV showed the numerically greatest improvement across AUC, IDI, and NRI.

**Table 3:** Comparative incremental predictive performance of age, MAP, and ePWV in the eICU-CRD cohort.

Model	Time-dependent AUC	*P*-value	Continuous IDI (95%CI)	*P*-value	Continuous NRI (95%CI)	*P*-value
Base	0.817					
Base + Age	0.823	<0.001	0.011 (0.001–0.021)	0.029	0.127 (0.051–0.203)	<0.001
Base + MAP	0.821	<0.001	0.008 (0.001–0.015)	0.033	0.109 (0.044–0.174)	<0.001
Base + ePWV	0.828	<0.001	0.014 (0.002–0.026)	0.024	0.162 (0.107–0.239)	<0.001

Base model: Gender, BMI, heart rate, albumin, neutrophil percentage, serum creatinine, BNP, hemoglobin, chronic kidney disease, atrial fibrillation, and STEMI.

### Subgroup analysis

Among ICU patients with AMI in the eICU-CRD cohort, subgroup analyses showed that the association between elevated ePWV and increased risk of new-onset HF was generally consistent across the prespecified subgroups of sex, BMI, hypertension status, and MI type (Supplementary Figure S5). Notably, in patients without PCAD, higher ePWV was significantly associated with an increased risk of new-onset HF, whereas this association did not reach statistical significance in patients with PCAD, with a significant interaction observed (*P* for interaction < 0.001). This suggests that the predictive value of ePWV may be relatively limited in this specific population. Similar findings were observed in the cohort from the Second Affiliated Hospital of Nanchang University. A significant interaction was observed only in the PCAD subgroup (*P* for interaction < 0.001), likewise suggesting that the predictive value of ePWV may be limited in patients with PCAD (Supplementary Figure S6).

### Sensitivity analyses

To reduce potential misclassification of admission-present HF as incident HF, we repeated the analysis after excluding HF diagnoses recorded within the first 24 h after ICU admission. A total of 4,358 patients remained, among whom 322 subsequently developed HF. The association remained significant after full adjustment (per 1-unit increase in ePWV: HR = 1.11, 95% CI: 1.05–1.16, *P* < 0.001). The highest ePWV tertile (HR = 1.79, 95% CI: 1.33–2.40, *P* < 0.001) and the high-ePWV group defined by the optimal cutoff (HR = 1.72, 95% CI: 1.37–2.16, *P* < 0.001) also remained significantly associated with HF risk, consistent with the primary analysis (Supplementary Table S9). In the competing-risk analysis, the association between ePWV and new-onset HF remained significant. After full adjustment, each 1-unit increase in ePWV was associated with a 17% higher subdistribution hazard of HF (sHR=1.17, 95% CI: 1.12–1.24, *P* < 0.001). Compared with T1, the sHRs were 1.55 (95% CI: 1.13–2.12, *P* = 0.008) for T2 and 2.02 (95% CI: 1.49–2.74, *P* < 0.001) for T3 (*P* for trend<0.001) (Supplementary Table S10). Similar results were observed after excluding HF events within the first 24 h. Further adjustment for vasoactive/inotropic drug use did not materially change the results (eICU-CRD: HR, 1.12; 95% CI, 1.07–1.17; *P* < 0.001; external cohort: OR, 1.16; 95% CI, 1.01–1.34; *P* = 0.042), with consistent categorical findings (Supplementary Table S11 and Supplementary Table S12).

## Discussion

In this study of ICU patients with AMI, higher ePWV was independently and nonlinearly associated with a greater risk of in-hospital new-onset HF and provided incremental predictive value beyond conventional clinical models. This association was generally consistent across subgroups stratified by hypertension status and myocardial infarction type, whereas its predictive value appeared to be limited in patients with PCAD.

Previous studies on arterial stiffness have mainly focused on the general population and consistently shown that, whether assessed by baPWV or ePWV, higher arterial stiffness is associated with an increased risk of new-onset HF. Zhang et al. reported that increased arterial stiffness, as reflected by baPWV, was independently associated with incident HF in the general population (9). Ji et al. further confirmed that elevated ePWV was also associated with a higher risk of new-onset HF and could improve risk reclassification (16). Heffernan et al. extended these findings by showing that higher ePWV was associated with a higher incidence of new-onset HF across different HF phenotypes (19). In patients with AMI, ePWV has also been linked to adverse outcomes other than new-onset HF. Xu et al. showed that ePWV was an independent predictor of long-term cardiovascular and all-cause mortality in patients with AMI (20), indicating its prognostic stratification value in this population. Kim et al. further reported that, among high-risk patients with coronary artery disease undergoing PCI, higher ePWV was associated with a greater risk of major adverse cardiovascular events (21). Even in relatively stable populations, such as patients with stable angina, ePWV has been associated with all-cause mortality, myocardial infarction, and stroke beyond traditional risk factors (22). However, direct evidence regarding more clinically specific endpoints, such as new-onset HF among ICU patients with AMI, remains limited. Our study therefore extends the available evidence on the prognostic value of ePWV to AMI patients requiring ICU care.

In this study, higher ePWV was associated with an increased risk of new-onset HF during hospitalization among ICU patients with AMI, consistent with previous studies (16). RCS analysis further showed a nonlinear positive association between ePWV and new-onset HF, without a clear threshold effect. In other words, the risk increase was modest at lower ePWV levels but became steeper at higher levels, suggesting that a simple linear model may not fully capture this relationship. These findings indicate that, among ICU patients with AMI, the risk reflected by ePWV increases progressively with higher ePWV levels. This pattern is biologically plausible and consistent with the progressive worsening of vascular aging and arterial stiffness, which continuously increases cardiac load (23). It also suggests that vascular aging-related risk may begin to affect prognosis at an earlier stage, rather than only when arterial stiffness becomes severe. Although patients who developed HF were older, ePWV should not be interpreted as a prognostic marker independent of age or MAP, as both are components of its equation. Incremental value analysis showed that adding age, MAP, and ePWV separately to the same clinical base model improved model performance in all three cases. Among these models, the addition of ePWV produced the numerically greatest improvements in AUC, IDI, and NRI compared with adding age or MAP alone. Because ePWV integrates age, MAP, nonlinear age terms, and age–MAP interaction terms within a predefined equation, it may be regarded as an alternative parameterization of age- and blood pressure-related vascular risk. Its apparent predictive advantage over age or MAP alone may therefore reflect a more effective integration of their joint and nonlinear effects. Accordingly, ePWV may serve as a convenient composite surrogate for risk stratification rather than an independent source of prognostic information beyond age and blood pressure. This interpretation is consistent with the view of Cheng et al., who suggested that ePWV may reflect arterial stiffness and its related risk more comprehensively than the original variables alone (24).

Subgroup analysis showed no marked difference in this association across hypertension status or myocardial infarction type, suggesting that ePWV may reflect a more general burden of vascular aging and arterial stiffness rather than the risk of a specific subtype. Notably, no significant predictive effect of ePWV was observed in patients with PCAD. One possible explanation is that patients with PCAD are younger by definition and have a relatively restricted age distribution, which may reduce the contribution of age-related vascular aging captured by ePWV. In addition, PCAD is more strongly influenced by genetic susceptibility and lipid-related abnormalities, including familial hypercholesterolemia, elevated lipoprotein(a), and dyslipidemia (25, 26). The prognostic value of ePWV may also vary across different comorbidity profiles. Conditions such as chronic kidney disease, atrial fibrillation, diabetes, and hypertension may influence arterial stiffness, hemodynamic load, and HF susceptibility, potentially modifying the risk information captured by ePWV. Although the association was generally consistent across the available subgroups in our study, further studies in patients with specific comorbidities and multimorbidity are warranted to better define the clinical applicability of ePWV. The external cohort showed a generally consistent direction of association with the primary eICU-CRD cohort, providing supportive evidence for the main findings. However, given the limited number of in-hospital HF events, estimates from extensively adjusted and subgroup models should be considered exploratory. The findings for new-onset HF within 1 year after discharge should also be interpreted cautiously as exploratory. Although the direction of the association still suggested that higher ePWV may be associated with a progressively increased risk of post-discharge HF, the lack of statistical significance indicates that this evidence should be regarded as a trend rather than a definitive conclusion. Possible explanations include the limited follow-up sample size, insufficient outcome events, and the influence of more post-discharge factors, such as subsequent treatment, lifestyle changes, quality of reperfusion, and baseline cardiac function, which may have reduced statistical power.

Although the mechanisms underlying the observed association between ePWV and new-onset HF among ICU patients with AMI are not yet fully understood, several explanations may be proposed. From a hemodynamic perspective, increased arterial stiffness leads to earlier return of reflected waves, resulting in elevated central systolic blood pressure, widened pulse pressure, and persistently increased left ventricular afterload, while reduced diastolic pressure may impair coronary perfusion and microvascular function (27, 28). At the cellular and molecular levels, vascular aging is characterized by endothelial dysfunction, vascular smooth muscle cell senescence and phenotypic switching, chronic inflammation, oxidative stress, and extracellular matrix remodeling (29, 30). Endothelial and smooth muscle cells in aged vessels may acquire a proinflammatory senescence-associated secretory phenotype, while increased reactive oxygen species and reduced nitric oxide bioavailability further aggravate arterial stiffness (31, 32). Inflammation, RAAS activation, and collagen deposition/fibrosis not only stiffen the arterial wall but may also affect the myocardium, thereby promoting myocardial hypertrophy, interstitial fibrosis, and diastolic dysfunction (33, 34). Animal studies support these observations: increased aortic stiffness in aged mice precedes myocardial hypertrophy and blood pressure abnormalities, suggesting that vascular stiffness may be an early upstream event (35), and arterial stiffness models such as Runx2-smTg mice also exhibit structural and functional cardiac abnormalities (36). Taken together, elevated ePWV may reflect not only age and blood pressure, but also the clinical manifestation of vascular aging-related pathological processes.

Nevertheless, this study has several limitations. First, ePWV is a surrogate index derived from age and blood pressure rather than a direct measure of arterial stiffness, such as cfPWV or baPWV. Second, as this was a multicenter cohort study, the diagnosis of HF could not be fully standardized across centers, which may have introduced some information bias. Given the limited size of the external validation cohort, these analyses should be considered supportive and exploratory, and larger independent ICU AMI cohorts are required for further validation. Although we excluded documented prior/admission HF and performed a sensitivity analysis excluding HF events within 24 h, some unrecognized pre-existing or admission-present HF may still have been misclassified as incident HF, and residual outcome misclassification cannot be completely excluded. In addition, ePWV was calculated from a single blood pressure measurement during hospitalization. Although we adjusted for vasoactive/inotropic drug use at the time of measurement, the temporal impact of subsequent changes during disease progression, therapeutic interventions, and hemodynamic fluctuations could not be evaluated. In addition, both the derivation and validation cohorts consisted of patients with AMI requiring ICU care, who likely represent a higher-risk and more clinically selected population than the overall AMI population. Therefore, the generalizability of our findings to patients with less severe AMI who do not require ICU admission may be limited, and further validation in broader AMI populations is warranted. Finally, although multiple potential confounders were adjusted for and sensitivity and subgroup analyses were performed, residual confounding cannot be completely excluded. Future prospective studies with repeated measurements and direct assessments of arterial stiffness are needed to further validate the clinical value of ePWV in patients with AMI.

## Conclusion

Among ICU patients with AMI, higher ePWV was independently and nonlinearly associated with an increased risk of in-hospital new-onset HF. As a simple surrogate marker of vascular aging and arterial stiffness-related risk, ePWV may aid early risk stratification in this high-risk population. Its prognostic value in broader AMI populations and for long-term post-discharge HF requires further validation in larger prospective cohorts.

## Data Availability

The original contributions presented in the study are included in the article/Supplementary Material, further inquiries can be directed to the corresponding author.
