# Supplementary material for: Estimated pulse wave velocity and risk of new-onset heart failure in ICU patients with acute myocardial infarction: a multicenter cohort study
Source: Front Med (Lausanne). 2026 Sep 22;13:1930499. doi: 10.3389/fmed.2026.1930499 (PMC13638576; doi:10.3389/fmed.2026.1930499)
Supplement: Supplementary file 1 [file Supplementaryfile1.docx]

**Supplementary File**

**Supplementary Tables**

**Supplementary Table S1.** Missingness of Baseline Study Variables	2

**Supplementary Table S2.** Quantitative Convergence Diagnostics for Multiple Imputation	3

**Supplementary Table S3.** ICD Codes for HF Diagnosis	4

**Supplementary Table S4.** Collinearity Analysis	6

**Supplementary Table S5.** Baseline Characteristics of the Study Population in the Second Affiliated Hospital of Nanchang University Cohort Stratified by Outcome	7

**Supplementary Table S6.** Association of ePWV With In-Hospital Heart Failure in the Nanchang University Second Hospital Cohort	9

**Supplementary Table S7.** Association of ePWV With Heart Failure Within 1 Year After Discharge in AMI Patients From the Nanchang University Second Hospital Cohort	10

**Supplementary Table S8.** Threshold Effect Analysis in the eICU-CRD Cohort	11

**Supplementary Table S9.** Association of ePWV With Heart Failure in the eICU-CRD Cohort After Excluding Very Early HF Events (Within 24 Hours of Admission)	12

**Supplementary Table S10.** Competing-risk Analysis of the Association Between ePWV and New-Onset Heart Failure in the eICU-CRD Cohort	13

**Supplementary Figures**

**Supplementary Figure S1.** Flowchart of Patient Selection	14

**Supplementary Figure S2.** Feature Selection by LASSO Regression	15

**Supplementary Figure S3.** Cumulative Risk Curves for HF in the eICU-CRD Cohort After Excluding Very Early HF Events (Within 24 Hours of Admission)	16

**Supplementary Figure S4.** HF Within 1 Year After Discharge in the External Validation Cohort	17

**Supplementary Figure S5.** Forest Plot of Subgroup Analysis in the eICU-CRD Cohort	18

**Supplementary Figure S6.** Forest Plot of Subgroup Analysis in the Nanchang University Second Hospital Cohort	19

**Supplementary Table S1. Missingness of baseline study variables**

| **Variable** | **Missing, n (%)** | **Imputation_status** |
| --- | --- | --- |
| Age | 0 (0%) | No |
| Male | 0 (0%) | No |
| Height | 673 (14.96%) | Yes |
| Weight | 467 (10.38%) | Yes |
| STEMI | 0 (0%) | No |
| PCAD | 0 (0%) | No |
| ePWV | 0 (0%) | No |
| Temperature | 0 (0%) | No |
| Heart rate | 0 (0%) | No |
| SBP | 0 (0%) | No |
| DBP | 0 (0%) | No |
| SpO2 | 0 (0%) | No |
| Respiratory rate | 0 (0%) | No |
| WBC | 49 (1.09%) | Yes |
| RBC | 49 (1.09%) | Yes |
| Platelets | 49 (1.09%) | Yes |
| Hemoglobin | 49 (1.09%) | Yes |
| Neutrophil percentage | 49 (1.09%) | Yes |
| Monocyte percentage | 49 (1.09%) | Yes |
| Lymphocyte percentage | 49 (1.09%) | Yes |
| Albumin | 255 (5.67%) | Yes |
| Creatinine | 53 (1.18%) | Yes |
| BUN | 53 (1.18%) | Yes |
| ALT | 56 (1.24%) | Yes |
| AST | 56 (1.24%) | Yes |
| Glucose | 0 (0%) | No |
| Triglycerides | 710 (15.78%) | Yes |
| Total cholesterol | 710 (15.78%) | Yes |
| LDL-C | 746 (16.59%) | Yes |
| HDL-C | 710 (15.78%) | Yes |
| Sodium | 0 (0%) | No |
| Potassium | 0 (0%) | No |
| BNP | 365 (8.11%) | Yes |
| CKD | 0 (0%) | No |
| AF | 0 (0%) | No |
| Hypertension | 0 (0%) | No |
| Diabetes mellitus | 0 (0%) | No |

**Supplementary Table S2. Quantitative convergence diagnostics for multiple imputation**

| **Variable** | **Standardized Between-chain Spread** | **Maximum Standardized Slope Over Final 5 Iterations** |
| --- | --- | --- |
| Height | 0.040 | 0.020 |
| Weight | 0.048 | 0.021 |
| WBC | 0.151 | 0.084 |
| RBC | 0.128 | 0.061 |
| Platelets | 0.161 | 0.058 |
| Hemoglobin | 0.134 | 0.074 |
| Neutrophil % | 0.172 | 0.090 |
| Monocyte % | 0.082 | 0.028 |
| Lymphocyte % | 0.111 | 0.052 |
| Albumin | 0.054 | 0.039 |
| Creatinine | 0.088 | 0.070 |
| BUN | 0.073 | 0.053 |
| ALT | 0.212 | 0.128 |
| AST | 0.194 | 0.081 |
| TG | 0.041 | 0.027 |
| TC | 0.026 | 0.028 |
| LDL-C | 0.037 | 0.025 |
| HDL-C | 0.025 | 0.015 |
| BNP | 0.039 | 0.028 |

***Standardized between-chain spread reflects between-chain variability relative to the observed standard deviation, while the late-iteration slope was used to assess residual systematic drift. Overall, no substantial between-chain separation or persistent late-iteration drift was observed.***

**Supplementary Table S3. ICD codes for HF diagnosis**

| **Icdcode** | **diagnosisstring** |
| --- | --- |
| 038.9, 428.0, R65.20, I50.9 | cardiovascular\|shock / hypotension\|sepsis\|sepsis with single organ dysfunction- congestive heart failure |
| 038.9, 428.0, R65.20, I50.9 | infectious diseases\|systemic/other infections\|sepsis\|sepsis with single organ dysfunction- congestive heart failure |
| 038.9, 428.0, R65.20, I50.9 | surgery\|infections\|sepsis\|sepsis with single organ dysfunction- congestive heart failure |
| 276.1, E87.1 | endocrine\|fluids and electrolytes\|hyponatremia\|due to congestive heart failure |
| 276.1, E87.1 | renal\|electrolyte imbalance\|hyponatremia\|due to congestive heart failure |
| 276.6, E87.70 | renal\|fluid imbalance\|hypervolemia\|in the absence of CHF |
| 276.6, E87.70 | renal\|fluid imbalance\|hypervolemia\|in the absence of renal failure and CHF |
| 404.91, I11.0 | cardiovascular\|vascular disorders\|hypertension\|with heart failure |
| 404.91, I11.0 | cardiovascular\|ventricular disorders\|hypertension\|with heart failure |
| 404.91, I11.0 | surgery\|acute cardiac problems\|hypertension\|with heart failure |
| 404.93, I13.2 | cardiovascular\|vascular disorders\|hypertension\|with heart failure and renal failure |
| 404.93, I13.2 | cardiovascular\|ventricular disorders\|hypertension\|with heart failure and renal failure |
| 415.0, I26.0 | cardiovascular\|ventricular disorders\|congestive heart failure\|cor pulmonale\|acute |
| 416.9, I27.9 | cardiovascular\|ventricular disorders\|congestive heart failure\|cor pulmonale\|chronic |
| 421.9, I33.9 | cardiovascular\|valvular disease\|valvular insufficiency\|with CHF\|acute (from endocarditis) |
| 422.90, I40.9 | cardiovascular\|infection\|myocarditis\|associated with CHF |
| 422.90, I40.9 | cardiovascular\|infection\|myocarditis\|unassociated with CHF |
| 428.0, I50.9 | cardiovascular\|ventricular disorders\|congestive heart failure |
| 428.0, I50.9 | cardiovascular\|ventricular disorders\|congestive heart failure\|biventricular |
| 428.0, I50.9 | cardiovascular\|ventricular disorders\|congestive heart failure\|decompensated |
| 428.0, I50.9 | cardiovascular\|ventricular disorders\|congestive heart failure\|right-sided |
| 428.0, I50.9 | cardiovascular\|ventricular disorders\|congestive heart failure\|right-sided\|due to left heart failure |
| 428.0, I50.9 | surgery\|acute cardiac problems\|congestive heart failure |
| 428.0, I50.9 | surgery\|acute cardiac problems\|congestive heart failure\|biventricular |
| 428.0, I50.9 | surgery\|acute cardiac problems\|congestive heart failure\|right-sided |
| 428.1, I50.1 | cardiovascular\|ventricular disorders\|congestive heart failure\|left-sided |
| 428.1, I50.1 | surgery\|acute cardiac problems\|congestive heart failure\|left-sided |
| 428.20, I50.20 | cardiovascular\|ventricular disorders\|congestive heart failure\|systolic |
| 428.21, I50.21 | cardiovascular\|ventricular disorders\|congestive heart failure\|systolic\|acute |
| 428.22, I50.22 | cardiovascular\|ventricular disorders\|congestive heart failure\|systolic\|chronic |
| 428.23, I50.23 | cardiovascular\|ventricular disorders\|congestive heart failure\|systolic\|acute on chronic |
| 428.30, I50.30 | cardiovascular\|ventricular disorders\|congestive heart failure\|diastolic |
| 428.31, I50.31 | cardiovascular\|ventricular disorders\|congestive heart failure\|diastolic\|acute |
| 428.32, I50.32 | cardiovascular\|ventricular disorders\|congestive heart failure\|diastolic\|chronic |
| 428.33, I50.33 | cardiovascular\|ventricular disorders\|congestive heart failure\|diastolic\|acute on chronic |
| 428.40, I50.40 | cardiovascular\|ventricular disorders\|congestive heart failure\|combined systolic and diastolic |
| 428.41, I50.41 | cardiovascular\|ventricular disorders\|congestive heart failure\|combined systolic and diastolic\|acute |
| 428.42, I50.42 | cardiovascular\|ventricular disorders\|congestive heart failure\|combined systolic and diastolic\|chronic |
| 428.43, I50.43 | cardiovascular\|ventricular disorders\|congestive heart failure\|combined systolic and diastolic\|acute on chronic |
| 511.9, J91.8 | pulmonary\|pleural disorders\|pleural effusion\|due to heart failure |
| 511.9, J91.8 | surgery\|cardiac surgery\|pleural effusion\|bilateral\|associated with CHF |
| 511.9, J91.8 | surgery\|cardiac surgery\|pleural effusion\|left\|associated with CHF |
| 511.9, J91.8 | surgery\|cardiac surgery\|pleural effusion\|right\|associated with CHF |
| 518.81, J96.00 | pulmonary\|respiratory failure\|acute respiratory failure due to volume overload without CHF |
| 518.84, J96.20 | pulmonary\|respiratory failure\|acute on chronic respiratory failure not due to CHF |
| 786.3, 428.1, I50.1, R04.9 | pulmonary\|disorders of vasculature\|pulmonary hemorrhage\|due to left sided heart failure |
| I50.9 | cardiovascular\|ventricular disorders\|congestive heart failure\|cor pulmonale |

**Supplementary Table S4. Collinearity Analysis**

| **Variable** | **GVIF** | **df** | **GVIF^(1/(2*df))** |
| --- | --- | --- | --- |
| ePWV | 1.12747723554674 | 1 | 1.0623312453779 |
| BMI | 1.0412144847962 | 1 | 1.02039917914324 |
| Sex | 1.05462073721291 | 1 | 1.02694729037712 |
| Heart rate | 1.14297832547165 | 1 | 1.06910164412541 |
| Albumin | 1.08932620824852 | 1 | 1.0437079132825 |
| Neutrophil percentage | 1.11367200171785 | 1 | 1.0553065913363 |
| Serum creatinine | 1.35512214049577 | 1 | 1.16409713533527 |
| BNP | 1.15974952072576 | 1 | 1.07691667306517 |
| Hemoglobin | 1.2534163691567 | 1 | 1.11956079297049 |
| Chronic kidney disease | 1.10372035731173 | 1 | 1.05058096180719 |
| Atrial fibrillation | 1.06684425808221 | 1 | 1.03288153148471 |
| ST-segment elevation myocardial infarction | 1.08693620375838 | 1 | 1.04256232607858 |

**Supplementary Table S5. Baseline Characteristics of the Study Population in the Second Affiliated Hospital of Nanchang University Cohort Stratified by Outcome**

| **Variables** | **Overall**  **(n = 255)** | **No new-onset HF**  **(n = 222)** | **New-onset HF**  **(n = 33)** | **P-value** |
| --- | --- | --- | --- | --- |
| Age, years | 66.8 ± 10.7 | 65.5 ± 9.7 | 75.7 ± 13.0 | < 0.001 |
| Male, n(%) | 159 (62.3) | 141 (63.5) | 18 (54.5) | 0.424 |
| Height, cm | 160.6 ± 8.4 | 161.3 ± 8.1 | 156.1 ± 9.2 | 0.001 |
| Weight, kg | 66.8 ± 9.6 | 67.3 ± 9.5 | 62.8 ± 9.9 | 0.012 |
| BMI, kg/m^2^ | 26.0 ± 4.1 | 26.0 ± 4.0 | 25.9 ± 4.7 | 0.924 |
| STEMI, n(%) | 152 (59.6) | 132 (59.4) | 20 (60.6) | 0.900 |
| PCAD, n(%) | 60 (23.5) | 56 (25.2) | 4 (12.1) | 0.098 |
| PCI, n(%) | 240 (94.12) | 215 (96.85) | 25 (75.76) | < 0.001 |
| Gensini, | 65.6 ± 34.4 | 63.6 ± 32.6 | 79.5 ± 42.7 | 0.013 |
| **Vital signs** |  |  |  |  |
| Temperature, ℃ | 36.5 ± 0.2 | 36.5 ± 0.2 | 36.5 ± 0.2 | 0.444 |
| Heart rate, beats/min | 80.7 ± 14.6 | 80.1 ± 14.7 | 84.8 ± 13.3 | 0.087 |
| SBP, mmhg | 128.1 ± 20.9 | 128.8 ± 20.5 | 123.2 ± 23.3 | 0.148 |
| DBP, mmhg | 78.6 ± 16.1 | 79.4 ± 15.6 | 73.4 ± 19.2 | 0.049 |
| MAP, mmhg | 91.55 ± 16.36 | 96.33 ± 18.99 | 90.84 ± 15.85 | 0.072 |
| SpO2, % | 96.0 ± 1.4 | 96.1 ± 1.4 | 95.2 ± 1.2 | 0.001 |
| Respiratory rate, beats/min | 19.1 ± 3.1 | 18.7 ± 2.8 | 21.9 ± 3.7 | < 0.001 |
| **Laboratory indicators** |  |  |  |  |
| WBC, k/uL | 10.2 ± 3.6 | 10.0 ± 3.5 | 11.3 ± 4.0 | 0.053 |
| RBC, k/uL | 4.3 ± 0.9 | 4.4 ± 0.9 | 3.7 ± 0.7 | < 0.001 |
| Platelets, k/uL | 218.9 ± 76.5 | 218.6 ± 73.9 | 220.5 ± 93.3 | 0.892 |
| Hemoglobin, g/L | 131.2 ± 26.6 | 133.7 ± 26.8 | 114.7 ± 18.3 | < 0.001 |
| Neu, % | 74.8 ± 11.6 | 74.3 ± 11.5 | 78.1 ± 12.5 | 0.078 |
| Mon, % | 6.8 ± 2.7 | 6.9 ± 2.8 | 6.1 ± 1.8 | 0.088 |
| Lym, % | 17.2 ± 10.2 | 17.5 ± 9.9 | 14.8 ± 11.6 | 0.159 |
| Albumin, mg/L | 36.7 ± 4.5 | 37.3 ± 4.0 | 33.0 ± 5.8 | < 0.001 |
| Creatinine, μmol/L | 119.8 ± 44.5 | 116.7 ± 53.2 | 140.4 ± 56.2 | 0.38 |
| BUN, mg/dL | 8.1 ± 4.8 | 7.5 ± 4.2 | 12.3 ± 6.5 | < 0.001 |
| ALT, U/L | 45.7 ±6.3 | 43.3 ± 6.9 | 61.7 ± 7.8 | 0.033 |
| AST, U/L | 154.3 ±18.9 | 157.7 ±17.0 | 131.2 ± 14.8 | 0.518 |
| Glucose, mmol/L | 7.1 ± 3.3 | 7.2 ± 3.2 | 6.7 ± 3.9 | 0.378 |
| Tg, mmol/L | 1.5 ± 0.9 | 1.6 ± 1.0 | 1.1 ± 0.3 | 0.004 |
| Tc, mmol/L | 4.3 ± 1.1 | 4.4 ± 1.0 | 3.2 ± 0.9 | < 0.001 |
| LDL, mmol/L | 2.6 ± 0.9 | 2.8 ± 0.8 | 1.7 ± 0.7 | < 0.001 |
| HDL, mmol/L | 1.0 ± 0.2 | 1.1 ± 0.2 | 1.0 ± 0.2 | 0.131 |
| Sodium, mmol/L | 136.8 ± 3.0 | 136.8 ± 3.0 | 137.2 ± 2.3 | 0.446 |
| Potassium, mmol/L | 3.9 ± 0.5 | 3.8 ± 0.5 | 4.0 ± 0.4 | 0.074 |
| BNP, pg/ml | 568.7 ± 124.3 | 393.8 ± 114.3 | 1744.9 ± 122.6 | < 0.001 |
| **Comorbidities, n (%)** |  |  |  |  |
| CKD | 91 (35.7) | 70 (31.5) | 21 (63.6) | < 0.001 |
| AF | 14 (5.5) | 11 (4.9) | 3 (9.1) | 0.573 |
| HTN | 170 (66.7) | 144 (64.8) | 26 (78.8) | 0.113 |
| DM | 84 (32.9) | 70 (31.5) | 14 (42.4) | 0.214 |

***BMI: body mass index, STEMI: ST-segment elevation myocardial infarction, PCAD: premature coronary artery disease, ePWV: estimated pulse wave velocity, SBP: systolic blood pressure, DBP: diastolic blood pressure,*** ***MAP: mean arterial pressure, SpO2: oxyhemoglobin saturation, WBC: white blood cell, RBC: red blood cell, BUN: blood urea nitrogen, ALT: alanine aminotransferase, AST: aspartate aminotransferase, Tg: triglyceride, Tc: total cholesterol, LDL: low-density lipoprotein, HDL: high-density lipoprotein, BNP: B-type natriuretic peptide, CKD: chronic kidney disease, AF: atrial fibrillation, HTN: hypertension, DM: diabetes mellitus,***

**Supplementary Table S6. Association of ePWV With In-Hospital Heart Failure in the Nanchang University Second Hospital Cohort**

| **ePWV** | **All** | **Events** | **Model 1** |  |  | **Model 2** |  |  | **Model 3** |  |
| --- | --- | --- | --- | --- | --- | --- | --- | --- | --- | --- |
|  |  |  | **OR (95% CI)** | ***P*-value** |  | **OR (95% CI)** | ***P*-value** |  | **OR (95% CI)** | ***P*-value** |
| Continuous | 255 | 33(12.9) | 1.22 (1.03-1.44) | P = 0.022 |  | 1.17 (1.02-1.36) | P = 0.031 |  | 1.16 (1.01-1.35) | P = 0.042 |
| Tertiles |  |  |  |  |  |  |  |  |  |  |
| T1（< 8.83） | 85 | 5 (5.9) | Ref. |  |  | Ref. |  |  | Ref. |  |
| T2（8.83- 10.84） | 85 | 10 (11.7) | 2.13 (0.70-6.53) | P = 0.184 |  | 2.11 (0.67-6.33) | P = 0.196 |  | 1.81 (0.58-5.64) | P = 0.307 |
| T3（> 10.84） | 85 | 18 (21.2) | 4.30 (1.52-12.19) | P = 0.006 |  | 4.12 (1.42-11.27) | P = 0.009 |  | 2.99 (1.05-8.81) | P = 0.041 |
| P for trend |  |  |  | P = 0.004 |  |  | P = 0.004 |  |  | P = 0.039 |
| Cutoff-based |  |  |  |  |  |  |  |  |  |  |
| Q1（≤ 10.42） | 158 | 13(8.4) | Ref. |  |  | Ref. |  |  | Ref. |  |
| Q2（> 10.42） | 97 | 20(20.6) | 2.48 (1.17-5.24) | P = 0.018 |  | 2.42 (1.15-5.17) | P = 0.021 |  | 2.21 (1.02-4.78) | P = 0.044 |

Model 1: unadjusted.

Model 2: adjusted for gender, BMI, and heart rate.

Model 3: adjusted for gender, BMI, heart rate, albumin, neutrophil percentage, creatinine, BNP, hemoglobin, CKD, AF, Gensini, PCI and STEMI.

**Supplementary Table S7. Association of ePWV With Heart Failure Within 1 Year After Discharge in AMI Patients From the Nanchang University Second Hospital Cohort**

| **ePWV** | **All** | **Events** | **Model 1** |  |  | **Model 2** |  |  | **Model 3** |  |
| --- | --- | --- | --- | --- | --- | --- | --- | --- | --- | --- |
|  |  |  | **HR (95% CI)** | ***P*-value** |  | **HR (95% CI)** | ***P*-value** |  | **HR (95% CI)** | ***P*-value** |
| Continuous | 246 | 17 (6.9) | 1.33 (1.02-1.73) | P = 0.034 |  | 1.31 (0.98-1.74) | P = 0.068 |  | 1.14 (0.80-1.62) | P = 0.470 |
| Tertiles |  |  |  |  |  |  |  |  |  |  |
| T1（< 8.72） | 81 | 3 (3.7) | Ref. |  |  | Ref. |  |  | Ref. |  |
| T2（8.72- 10.57） | 82 | 4 (4.9) | 1.39 (0.31-6.21) | P = 0.667 |  | 1.39 (0.32-6.24) | P = 0.678 |  | 0.82 (0.16-4.28) | P = 0.810 |
| T3（> 10.57） | 83 | 10 (12.0) | 4.03 (1.09-14.84) | P = 0.036 |  | 4.05 (1.07-15.30) | P = 0.039 |  | 2.75 (0.66-11.44) | P = 0.159 |
| P for trend |  |  |  | P = 0.026 |  |  | P = 0.042 |  |  | P = 0.211 |
| Cutoff-based |  |  |  |  |  |  |  |  |  |  |
| Q1（≤ 10.12） | 160 | 7 (4.4) | Ref. |  |  | Ref. |  |  | Ref. |  |
| Q2（> 10.12） | 86 | 10 (11.6) | 2.76 (1.05-7.25) | P = 0.039 |  | 2.44 (0.83-7.22) | P = 0.107 |  | 2.04 (0.64-6.52) | P = 0.228 |

Model 1: unadjusted.

Model 2: adjusted for gender, BMI, and heart rate.

Model 3: adjusted for gender, BMI, heart rate, albumin, neutrophil percentage, creatinine, BNP, hemoglobin, CKD, AF, Gensini, PCI and STEMI.

**Supplementary Table S8. Threshold Effect Analysis in the eICU-CRD Cohort**

| **Threshold effect analysis** | **Adjusted HR (95% CI)** | ***P*-value** |
| --- | --- | --- |
| ePWV (per 1 unit) |  |  |
| Total | 1.13 (1.09-1.18) | < 0.001 |
| Estimated threshold |  |  |
| < 10.54 | 1.06 (0.97-1.17) | 0.216 |
| ≥ 10.54 | 1.18 (1.10-1.27) | < 0.001 |
| P for likelihood ratio test |  | 0.142 |

Adjusted for gender, BMI, heart rate, albumin, neutrophil percentage, creatinine,BNP, hemoglobin, CKD, AF, and STEMI.。

**Supplementary Table S9. Association of ePWV with heart failure in the eICU‑CRD cohort after excluding very early HF events (within 24 hours of admission)**

| **ePWV** | **All** | **Events** | **Model 1** |  |  | **Model 2** |  |  | **Model 3** |  |
| --- | --- | --- | --- | --- | --- | --- | --- | --- | --- | --- |
|  |  |  | **HR (95% CI)** | ***P*-value** |  | **HR (95% CI)** | ***P*-value** |  | **HR (95% CI)** | ***P*-value** |
| **After 24 hours** |  |  |  |  |  |  |  |  |  |  |
| Continuous | 4358 | 322 (7.3) | 1.17 (1.12-1.23) | P < 0.001 |  | 1.16 (1.11-1.22) | P < 0.001 |  | 1.11 (1.05-1.16) | P < 0.001 |
| Tertiles |  |  |  |  |  |  |  |  |  |  |
| T1（< 8.90） | 1454 | 66 (4.5) | Ref. |  |  | Ref. |  |  | Ref. |  |
| T2（8.90- 10.90） | 1452 | 102 (7.0) | 1.56 (1.15-2.13) | P = 0.005 |  | 1.57 (1.15-2.14) | P = 0.004 |  | 1.50 (1.10-2.05) | P = 0.011 |
| T3（> 10.90） | 1452 | 154 (10.6) | 2.42 (1.82-3.23) | P < 0.001 |  | 2.34 (1.75-3.13) | P < 0.001 |  | 1.79 (1.33-2.40) | P < 0.001 |
| P for trend |  |  |  | P < 0.001 |  |  | P < 0.001 |  |  | P < 0.001 |
| Cutoff-based |  |  |  |  |  |  |  |  |  |  |
| Q1（≤ 10.54） | 2678 | 136 (5.0) | Ref. |  |  | Ref. |  |  | Ref. |  |
| Q2（> 10.54） | 1680 | 186 (11.0) | 2.26 (1.81-2.82) | P < 0.001 |  | 2.19 (1.76-2.74) | P < 0.001 |  | 1.72 (1.37-2.16) | P < 0.001 |

**Model 1: unadjusted.**

**Model 2: adjusted for gender, BMI, and heart rate.**

**Model 3: adjusted for gender, BMI, heart rate, albumin, neutrophil percentage, creatinine, BNP, hemoglobin, CKD, AF, and STEMI.**

**Supplementary Table S10. Competing-risk Analysis of the Association Between ePWV and New-Onset Heart Failure in the eICU-CRD Cohort**

| **ePWV** | **All** | **Events** | **Model 1** |  |  | **Model 2** |  |  | **Model 3** |  |
| --- | --- | --- | --- | --- | --- | --- | --- | --- | --- | --- |
|  |  |  | **sHR (95% CI)** | ***P*-value** |  | **sHR (95% CI)** | ***P*-value** |  | **sHR (95% CI)** | ***P*-value** |
| **In-hospital** |  |  |  |  |  |  |  |  |  |  |
| Continuous | 4498 | 462(10.2) | 1.25 (1.19-1.31) | P < 0.001 |  | 1.24 (1.18-1.30) | P < 0.001 |  | 1.17 (1.12-1.24) | P < 0.001 |
| Tertiles |  |  |  |  |  |  |  |  |  |  |
| T1（< 8.93） | 1499 | 92 (6.1) | Ref. |  |  | Ref. |  |  | Ref. |  |
| T2（8.93- 10.98） | 1499 | 149 (9.9) | 1.79 (1.32-2.44) | P < 0.001 |  | 1.78 (1.30-2.43) | P = 0.001 |  | 1.55 (1.13-2.12) | P = 0.008 |
| T3（> 10.98） | 1500 | 221 (14.7) | 2.87 (2.15-3.84) | P < 0.001 |  | 2.71 (2.02-3.63) | P < 0.001 |  | 2.02 (1.49-2.74) | P < 0.001 |
| P for trend |  |  |  | P < 0.001 |  |  | P < 0.001 |  |  | P < 0.001 |
| Cutoff-based |  |  |  |  |  |  |  |  |  |  |
| Q1（≤ 10.54） | 2723 | 181(6.6) | Ref. |  |  | Ref. |  |  | Ref. |  |
| Q2（> 10.54） | 1775 | 281(15.8) | 2.82 (2.27-3.52) | P < 0.001 |  | 2.74 (2.20-3.43) | P < 0.001 |  | 2.20 (1.75-2.77) | P < 0.001 |
| **After 24 hours** |  |  |  |  |  |  |  |  |  |  |
| Continuous | 4358 | 322 (7.3) | 1.16 (1.11-1.22) | P < 0.001 |  | 1.15 (1.09-1.21) | P < 0.001 |  | 1.09 (1.04-1.15) | P < 0.001 |
| Tertiles |  |  |  |  |  |  |  |  |  |  |
| T1（< 8.90） | 1454 | 66 (4.5) | Ref. |  |  | Ref. |  |  | Ref. |  |
| T2（8.90- 10.90） | 1452 | 102 (7.0) | 1.48 (1.15-1.99) | P = 0.009 |  | 1.35 (1.12-1.72) | P = 0.014 |  | 1.23 (1.09-1.48) | P = 0.027 |
| T3（> 10.90） | 1452 | 154 (10.6) | 2.24 (1.67-2.99) | P < 0.001 |  | 2.14 (1.59-2.86) | P < 0.001 |  | 1.62 (1.20-2.20) | P < 0.001 |
| P for trend |  |  |  | P < 0.001 |  |  | P < 0.001 |  |  | P < 0.001 |
| Cutoff-based |  |  |  |  |  |  |  |  |  |  |
| Q1（≤ 10.54） | 2678 | 136 (5.0) | Ref. |  |  | Ref. |  |  | Ref. |  |
| Q2（> 10.54） | 1680 | 186 (11.0) | 2.20 (1.74-2.77) | P < 0.001 |  | 2.16 (1.70-2.74) | P < 0.001 |  | 1.74 (1.36-2.22) | P < 0.001 |

**Model 1: unadjusted.**

**Model 2: adjusted for gender, BMI, and heart rate.**

**Model 3: adjusted for gender, BMI, heart rate, albumin, neutrophil percentage, creatinine, BNP, hemoglobin, CKD, AF, and STEMI.**

**After 24 hours: Sensitivity analysis excluding HF diagnoses within the first 24 h**

**Competing events were defined as discharge, transfer, or death occurring before new-onset HF. sHRs were estimated using Fine–Gray subdistribution hazard models. sHR, subdistribution hazard ratio.**

**Supplementary Table S11. The association of ePWV with in-hospital new-onset heart failure in critically ill AMI patients in the eICU-CRD cohort after further adjustment for Vasoactive/inotropic drugs.**

| **ePWV** | **All** | **Events** | **Model 1** |  |  | **Model 2** |  |  | **Model 3** |  |
| --- | --- | --- | --- | --- | --- | --- | --- | --- | --- | --- |
|  |  |  | **HR (95% CI)** | ***P*-value** |  | **HR (95% CI)** | ***P*-value** |  | **HR (95% CI)** | ***P*-value** |
| **In-hospital** |  |  |  |  |  |  |  |  |  |  |
| Continuous | 4498 | 462(10.2) | 1.22 (1.17-1.27) | P < 0.001 |  | 1.20 (1.15-1.25) | P < 0.001 |  | 1.12 (1.07-1.17) | P < 0.001 |
| Tertiles |  |  |  |  |  |  |  |  |  |  |
| T1（< 8.93） | 1499 | 92 (6.1) | Ref. |  |  | Ref. |  |  | Ref. |  |
| T2（8.93- 10.98） | 1499 | 149 (9.9) | 1.63 (1.24-2.14) | P < 0.001 |  | 1.60 (1.22-2.10) | P = 0.001 |  | 1.40 (1.09-1.80) | P = 0.008 |
| T3（> 10.98） | 1500 | 221 (14.7) | 2.50 (1.94-3.22) | P < 0.001 |  | 2.33 (1.80-3.00) | P < 0.001 |  | 1.65 (1.23-2.14) | P < 0.001 |
| P for trend |  |  |  | P < 0.001 |  |  | P < 0.001 |  |  | P < 0.001 |
| Cutoff-based |  |  |  |  |  |  |  |  |  |  |
| Q1（≤ 10.54） | 2723 | 181(6.6) | Ref. |  |  | Ref. |  |  | Ref. |  |
| Q2（> 10.54） | 1775 | 281(15.8) | 2.56 (2.11-3.11) | P < 0.001 |  | 2.44 (2.01-2.97) | P < 0.001 |  | 1.81 (1.42-2.18) | P < 0.001 |

**Model 1: unadjusted.**

**Model 2: adjusted for gender, BMI, and heart rate.**

**Model 3: adjusted for gender, BMI, heart rate, albumin, neutrophil percentage, creatinine, BNP, hemoglobin, CKD, AF, STEMI and Vasoactive/inotropic drugs.**

**Vasoactive/inotropic drug use before or at the first non-invasive blood pressure measurement occurred in 157/4,498 patients (3.5%), including 35/462 (7.6%) with HF and 122/4,036 (3.0%) without HF.**

**Supplementary Table S12. Association of ePWV with in-hospital heart failure in the Nanchang University Second Hospital cohort after further adjustment for vasoactive drugs.**

| **ePWV** | **All** | **Events** | **Model 1** |  |  | **Model 2** |  |  | **Model 3** |  |
| --- | --- | --- | --- | --- | --- | --- | --- | --- | --- | --- |
|  |  |  | **OR (95% CI)** | ***P*-value** |  | **OR (95% CI)** | ***P*-value** |  | **OR (95% CI)** | ***P*-value** |
| Continuous | 255 | 33(12.9) | 1.22 (1.03-1.44) | P = 0.022 |  | 1.17 (1.02-1.36) | P = 0.031 |  | 1.16 (1.01-1.34) | P = 0.042 |
| Tertiles |  |  |  |  |  |  |  |  |  |  |
| T1（< 8.83） | 85 | 5 (5.9) | Ref. |  |  | Ref. |  |  | Ref. |  |
| T2（8.83- 10.84） | 85 | 10 (11.7) | 2.13 (0.70-6.53) | P = 0.184 |  | 2.11 (0.67-6.33) | P = 0.196 |  | 1.81 (0.58-5.64) | P = 0.308 |
| T3（> 10.84） | 85 | 18 (21.2) | 4.30 (1.52-12.19) | P = 0.006 |  | 4.12 (1.42-11.27) | P = 0.009 |  | 2.97 (1.04-8.81) | P = 0.044 |
| P for trend |  |  |  | P = 0.004 |  |  | P = 0.004 |  |  | P = 0.039 |
| Cutoff-based |  |  |  |  |  |  |  |  |  |  |
| Q1（≤ 10.42） | 158 | 13(8.4) | Ref. |  |  | Ref. |  |  | Ref. |  |
| Q2（> 10.42） | 97 | 20(20.6) | 2.48 (1.17-5.24) | P = 0.018 |  | 2.42 (1.15-5.17) | P = 0.021 |  | 2.19 (1.02-4.67) | P = 0.043 |

**Model 1: unadjusted.**

**Model 2: adjusted for gender, BMI, and heart rate.**

**Model 3: adjusted for gender, BMI, heart rate, albumin, neutrophil percentage, creatinine, BNP, hemoglobin, CKD, AF, Gensini, PCI, STEMI and Vasoactive/inotropic drugs.**

**Vasoactive/inotropic drug use before or at the first non-invasive blood pressure measurement occurred in 13/255 patients (5.1%), including 3/33 (9.1%) with HF and 10/222 (4.5%) without HF.**

**Supplementary Figure S1. Flowchart of Patient Selection**

**Supplementary Figure S2. Feature Selection by LASSO Regression**

**Supplementary Figure S3. Cumulative risk curves for HF in the eICU-CRD cohort after excluding very early HF events (within 24 hours of admission)**

**Supplementary Figure S4. HF Within 1 Year After Discharge in the External Validation Cohort**

**Supplementary Figure S5. Forest Plot of Subgroup Analysis in the eICU-CRD Cohort**

**Supplementary Figure S6. Forest Plot of Subgroup Analysis in the Nanchang University Second Hospital Cohort**
